# Extended left hepatectomy with sequential triple-vessel resection and reconstruction for advanced cholangiocarcinoma: a case report

**DOI:** 10.1093/jscr/rjag489

**Published:** 2026-06-19

**Authors:** Evonne Zoe Younan, Mansha Jiwane, Harinder Kaur Bains, Michael D Crawford, Charbel Sandroussi, Jerome Martin Laurence

**Affiliations:** Department of Hepatobiliary and Upper GI Surgery, Royal Prince Alfred Hospital, 50 Missenden Road, Camperdown, NSW, 2050, Australia; Department of Hepatobiliary and Upper GI Surgery, Royal Prince Alfred Hospital, 50 Missenden Road, Camperdown, NSW, 2050, Australia; Department of Hepatobiliary and Upper GI Surgery, Royal Prince Alfred Hospital, 50 Missenden Road, Camperdown, NSW, 2050, Australia; Department of Hepatobiliary and Upper GI Surgery, Royal Prince Alfred Hospital, 50 Missenden Road, Camperdown, NSW, 2050, Australia; Faculty of Medicine and Health, The University of Sydney, Camperdown, NSW, 2050, Australia; RPA Institute of Academic Surgery (IAS), 45 Missenden Road, Camperdown, NSW, 2050, Australia; Department of Hepatobiliary and Upper GI Surgery, Royal Prince Alfred Hospital, 50 Missenden Road, Camperdown, NSW, 2050, Australia; Faculty of Medicine and Health, The University of Sydney, Camperdown, NSW, 2050, Australia; RPA Institute of Academic Surgery (IAS), 45 Missenden Road, Camperdown, NSW, 2050, Australia; Department of Hepatobiliary and Upper GI Surgery, Royal Prince Alfred Hospital, 50 Missenden Road, Camperdown, NSW, 2050, Australia; Faculty of Medicine and Health, The University of Sydney, Camperdown, NSW, 2050, Australia; RPA Institute of Academic Surgery (IAS), 45 Missenden Road, Camperdown, NSW, 2050, Australia

**Keywords:** cholangiocarcinoma, hepatectomy, portal vein reconstruction, hepatic artery reconstruction, IVC reconstruction

## Abstract

The resectability of cholangiocarcinoma is often limited by vascular involvement; however, advancements in hepatobiliary and transplant surgery have enabled increasingly complex resections. We describe a novel, sequential approach to resection of cholangiocarcinoma involving triple-vessel reconstruction of the hepatic artery, portal vein, and inferior vena cava (IVC). A 71-year-old woman underwent resection of an intra-hepatic cholangiocarcinoma invading the hilar vessels. The procedure involved sequential resection, reconstruction and revascularization of the hepatic artery, portal vein and IVC before removing the specimen *en-bloc*. This approach preserved vascular inflow or outflow at each step, thereby minimizing the risk of ischemic liver injury and circumventing the time limitations of other techniques such as total vascular exclusion. The patient’s postoperative course was uneventful, and she was discharged on postoperative day 14. At 44-month follow-up, she remains well with no radiological evidence of recurrence. Sequential vascular resection may represent a feasible strategy for selected patients with locally advanced cholangiocarcinoma.

## Introduction

Resection of locally advanced cholangiocarcinoma remains one of the highest-risk elective procedures in liver surgery. The disease process is aggressive with significant peri-operative morbidity and mortality [1]. Assessment of resectability is primarily determined by the extent of vascular involvement, assuming there is adequate future liver remnant to prevent post-hepatectomy liver failure (PHLF) [2–4]. Vascular resection combined with hepatectomy poses a distinct challenge due to the liver ischaemia associated with temporary interruption of the blood flow the liver and inferior vena cava (IVC). While ischaemia of up to 60 min under normothermic conditions may be tolerated, there is no categorical ‘safe’ limit [5]. Moreover, morbidity and mortality increase incrementally in a non-linear fashion with ischaemia such that minimization of iscahemia should be the dominant strategy [6–8]. One solution to this challenge involves strategies adapted from transplantation, involving *in-situ* or *ex-situ* hypothermia and shunting. This case represents an alternative novel approach for the management of advanced cholangiocarcinoma involving sequential vascular resection and reconstruction of the hepatic arterial, portal venous and caval systems without any ischaemia.

## Case presentation

A 71-year-old otherwise healthy woman presented with incidental non-contrast computed tomography (CT) scan findings of a possible liver mass with left ductal dilatation. Due to anaphylaxis to iodinated contrast, a magnetic resonance imaging (MRI) scan with Primovist was performed, which demonstrated a 5 cm mass causing obstruction of segment 2 and 3 ducts, without evidence of involvement of the right portal vein, right hepatic artery or right lobe of the liver (Fig. 1, Supplemental Video 2). However, due to the imaging limitations, the true extent of her disease was not apparent prior to surgery. At exploration, her disease was found to invade the portal vein confluence, the right and left hepatic arterial branches and the IVC. The procedure was performed by sequential resection, reconstruction and revascularization so that the liver was never without arterial inflow and portal inflow simultaneously (Supplemental Video 1). The right lobe of the liver was mobilized from the IVC, except for an area of invasion of the tumor at the junction of the caudate lobe and para-caval portion of the caudate. Parenchymal transection was undertaken ~1 cm to the right of Cantlie’s line between the middle and right hepatic veins onto the IVC, leaving the caudate attached to the IVC due to invasion. Transection was performed without remnant liver inflow or outflow control and total blood loss was ~400 mL. The distal bile duct was then transected at the level of the superior border of the pancreas. The right hepatic duct was divided to the right of the tumor mass, revealing the right anterior and posterior sectoral ducts. Then the proper hepatic artery and the right hepatic artery were clamped and resection of the left hepatic artery accomplished by dividing the right hepatic artery just distal to its origin and proper hepatic artery proximal to left hepatic artery origin. Then end-to-end anastomosis of the proper hepatic artery to the right hepatic artery was performed and the hepatic artery was revascularized (Supplemental Video 2). Portal flow was preserved during arterial resection. Next, the main portal vein and right portal vein were clamped, divided proximal and distal to the left portal vein origin, and anastomosed with continuous 6/0 prolene (Supplemental Video 2). The portal inflow was restored. Finally, the IVC resection and reconstruction was performed preserving right hepatic venous outflow. The middle and left hepatic vein insertions to the IVC were divided and the IVC was clamped at the level below the right hepatic vein and below the area of tumor invasion. The involved IVC was then resected en-bloc with the specimen. Finally, the resected IVC segment was reconstructed using a tubularized bovine pericardium interposition graft (Supplemental Video 2). Roux-en-Y hepaticojejunostomy was then performed. The patient was admitted to the intensive care unit (ICU) where her post-operative course was unremarkable, requiring transfusion of one unit of blood. Transaminases peaked on post-operative day one, normalizing by the date of discharge (Fig. 2). Serum bilirubin was mildly elevated to 28 μmol/L and international normalised ratio (INR) was 1.3 on post-operative day zero and both normalized within 24–48 h. Her ICU length-of-stay was 120 hours and she was discharged after 14 days. Histopathology demonstrated a moderately differentiated pT3N1 tumor with perineural and vascular invasion into the left portal vein and outer wall of the IVC with sparing of the hepatic artery. Tumor was seen microscopically at the bile duct and portal vein margin (R1) and one of thirteen lymph nodes was positive. She was discharged on apixaban 5 mg twice daily. She completed adjuvant chemotherapy with capecitabine followed by adjuvant radiotherapy and remains well at last follow-up of 40 months with no evidence of recurrence on non-contrast CT.

**Figure 1:**
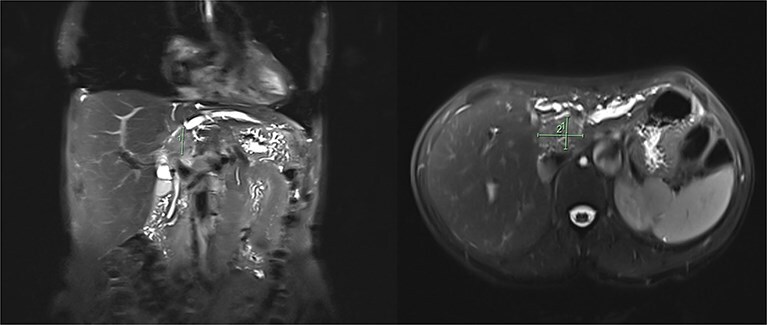
Pre-operative MRI liver was performed (due to known anaphylaxis to iodinated CT contrast) demonstrating a heterogenous soft tissue mass within the liver with obstruction of the segment 2 and 3 bile ducts.

**Figure 2:**
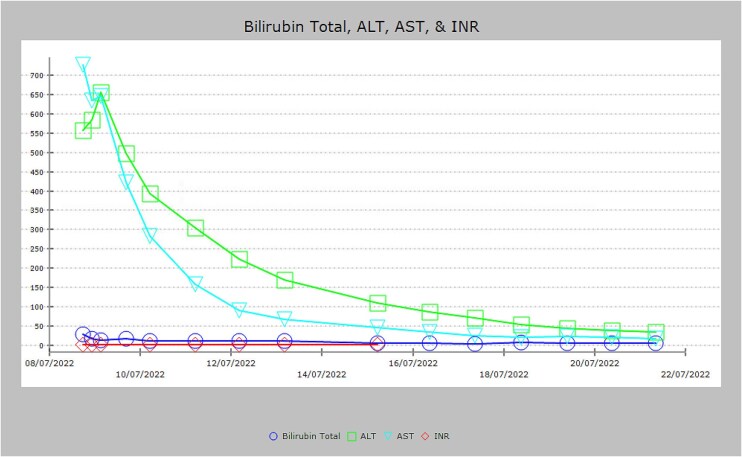
Graph demonstrating serum bilirubin, INR, and transaminases post-hepatectomy. Serum bilirubin and INR remained low throughout admission and peaked at 28 μmol/L and 1.3, respectively. ALT and AST peaked immediately post-operatively but down-trended before normalizing by time of discharge.

## Discussion

This case demonstrates a novel approach to the resection of advanced cholangiocarcinoma with involvement of major vasculature while minimizing ischaemic injury to the liver. By sequentially resecting and reconstructing the hepatic artery, portal vein and inferior vena cava while preserving the remaining vascular inflow and outflow at each stage, continuous hepatic perfusion was maintained throughout the procedure. To our knowledge, this exact stepwise preservation of the other two vascular systems during each stage of reconstruction has not been formally described in the literature. Hepatectomy with single vessel resection is well established in the management of locally advanced cholangiocarcinoma, with portal vein resections performed in 20%–40% of cases and reconstructed using either end-to-end anastomosis or interposition grafts [9]. Combined vascular resections are infrequently reported, and case reports have demonstrated acceptable outcomes of combined portal vein and hepatic artery reconstruction following hilar resection [10]. Alternative techniques for complex vascular involvement include total vascular exclusion (TVE) in which both the portal inflow and outflow are temporarily clamped. This approach is generally considered safe to an extent of 60 min in patients with normal liver parenchyma, limiting the time available for reconstruction [7]. Beyond two-vessel involvement, techniques adapted from liver transplantation surgery are generally applied. These includes *in-situ* liver perfusion and hypothermia, *ex-situ* and *ante-situm* approaches and more recently *in-situ* hypothermic oxygenated machine perfusion (HOPE) [11–13]. All of these, with the exception of the small reported number of HOPE cases, are associated with significant morbidity and mortality risk [13]. In contrast, the approach described in this case maintains continuous hepatic inflow and outflow throughout the operation, potentially reducing the risk of ischaemic liver injury while avoiding the physiological stress associated with total vascular exclusion or ex-situ techniques. It also avoids the complexity of HOPE and can be accomplished in non-transplant centers.

## Conclusion

This case demonstrates that management of advanced cholangiocarcinoma involving the hepatic arterial, portal venous and caval systems can be achieved using a sequential resection and reconstruction strategy that preserves continuous hepatic inflow and outflow, minimizing ischaemic liver injury. This approach may expand surgical options for locally advanced disease, although further experience is required to determine its reproducibility and long-term oncological outcomes.

## Supplementary Material

SupplementaryVideo1_rjag489

Supplementary2_rjag489
